# Buoyant hydrous mantle plume from the mantle transition zone

**DOI:** 10.1038/s41598-019-43103-y

**Published:** 2019-04-25

**Authors:** Takeshi Kuritani, Qun-Ke Xia, Jun-Ichi Kimura, Jia Liu, Kenji Shimizu, Takayuki Ushikubo, Dapeng Zhao, Mitsuhiro Nakagawa, Shumpei Yoshimura

**Affiliations:** 1 Graduate School of Science, Hokkaido University, Sapporo, Japan; 2 School of Earth Sciences, Zhejiang University, Hangzhou, China; 3 Department of Solid Earth Geochemistry, Japan Agency for Marine-Earth Science and Technology, Yokosuka, Japan; 4 Key Laboratory of Submarine Geosciences, Second Institute of Oceanography, State Oceanic Administration, Hangzhou, China; 5 Kochi Institute for Core Sample Research, Japan Agency for Marine-Earth Science and Technology, Nankoku, Japan; 6 Graduate School of Science, Tohoku University, Sendai, Japan

**Keywords:** Geochemistry, Petrology

## Abstract

Magmatism at some intraplate volcanoes and large igneous provinces (LIPs) in continental areas may originate from hydrous mantle upwelling (i.e. a plume) from the mantle transition zone (MTZ) at 410–660 km depths in the Earth’s deep interior. However, the ultimate origin of the magmatism, i.e. why mantle plumes could have been generated at the MTZ, remains unclear. Here, we study the buoyancy of a plume by investigating basalts from the Changbaishan volcano, beneath which a mantle plume from the hydrous MTZ is observed via seismology. Based on carefully determined water contents of the basalts, the potential temperature of the source mantle is estimated to be 1310–1400 °C, which is within the range of the normal upper mantle temperature. This observation suggests that the mantle plume did not have a significant excess heat, and that the plume upwelled because of buoyancy resulting from water supplied from the Pacific slab in the MTZ. Such a hydrous mantle plume can account for the formation of extremely hydrous LIP magmatism. The water was originally sourced from a stagnant slab and stored in the MTZ, and then upwelled irrespective of the presence or absence of a deep thermal plume.

## Introduction

Mantle plumes associated with magmatism at hotspots and large igneous provinces (LIPs) are commonly thought to be driven by thermal buoyancy originating from the steep thermal gradient at the core–mantle boundary^1,2^, as evidenced from their high mantle potential temperature^3–6^. In addition to this deeply rooted thermal plume model, there is growing evidence that some mantle plumes are generated from the hydrous mantle transition zone (MTZ) at 410–660 km depth that borders the upper and lower mantles. These examples are always large intraplate volcanoes^7^, flood basalts^8^, and LIPs^9^ in continental areas. Effusions of large amounts of volatile-rich magmas on-land must have caused prominent gas emission and severely impacted the Earth’s surface, which led to mass extinction in some cases^10^. Despite their significance in the Earth sciences, it remains unclear why such mantle plumes developed at the MTZ beneath continents. Water is clearly a plausible source for the buoyancy of a hydrous mantle plume^9,11,12^. However, whether the plume generation resulted solely from compositional buoyancy, or additional thermal buoyancy was required, has not been critically evaluated for any mantle plumes that evidently derived from the MTZ. Ancient magmatism is a less suitable target to evaluate buoyancy as we lack seismic data regarding mantle velocities.

**Figure 1:**
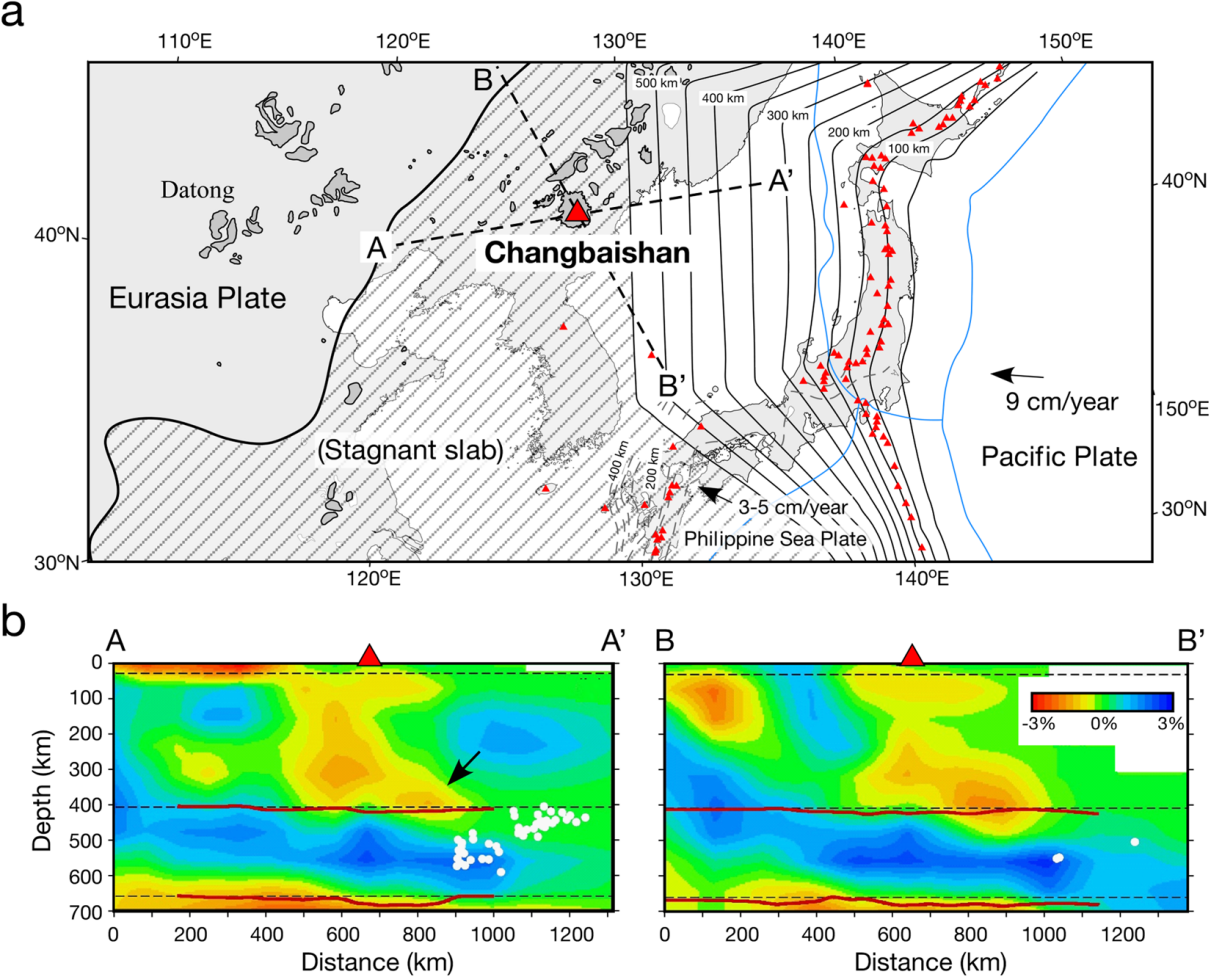
(**a**) Map showing the tectonic background of Northeast Asia. The red triangles denote active volcanoes. The grey patches denote representative Cenozoic volcanic fields in China^50^. The solid and dashed contour lines denote depths to the upper boundaries of the subducting Pacific and Philippine Sea slabs, respectively^51^. The blue lines denote plate boundaries at the surface. The shaded area indicates the range of the stagnant Pacific slab at depths > ~550 km in the mantle transition zone^52^. (**b**) Vertical cross-sections of P-wave tomography along two profiles shown in (**a**) passing through the Changbaishan volcano^7,53^. The red and blue colours denote the low and high P-wave velocity perturbations, respectively, whose scale (in %) is shown in cross section B–B’. The three dashed lines denote the Moho, 410-km, and 660-km discontinuities. The red lines represent geometries of the 410-km and 660-km discontinuities estimated from teleseismic receiver-functions^53^. The white dots denote deep earthquakes in the Pacific slab.

Changbaishan is among the best suited volcanoes for the study of a possible mantle plume from the hydrous MTZ. This volcano, located on the border between China and North Korea, is a continental intraplate volcano forming one of the largest active volcanic fields in Northeast Asia (Fig. 1). The volcanism has been sustained for >20 Myrs^13^ and has produced a basaltic lava plateau covering ~12,000 km^2^ ^14^. A caldera-forming eruption that occurred during the 10^th^ century was among the largest eruptions on Earth during the past 2,000 years^13^. Seismological studies have shown that the subducting Pacific slab is stagnant in the MTZ beneath the entire area of Eastern China^7,15^ (Fig. 1). The MTZ here is considerably hydrous in comparison to the globally dry MTZ as shown by electrical conductivity observations^16,17^. Beneath Changbaishan, a prominent low-velocity anomaly with a plume-like shape is observed across the upper mantle using P-wave seismic tomography^7^ (Fig. 1), which is among the most clearly imaged examples of a mantle plume originating from the MTZ. Geochemical studies of the basalts also show involvement of the stagnant slab and metasomatised peridotite materials from the MTZ in their mantle source^18,19^, yet water content in the mantle source has not been accurately determined. In this study, the water content of the basalt was determined using thermodynamic analyses and mineral melt inclusions for selected young basaltic samples. We use such measured water contents to estimate mantle potential temperatures using two independent models^20,21^, and we finally examine the source of the buoyancy of the mantle plume beneath the Changbaishan volcano.

## Results

The products of the Changbaishan volcano exhibit a wide compositional range from basalt to rhyolite (46–75 wt.% SiO_2_), with scarce andesitic lavas (Supplementary Fig. S1). During the activity of the volcano, basaltic magmas have been most common, although felsic magmas have dominated since ~3 Ma^13^. The basaltic magmas are primarily alkaline, but some tholeiitic (subalkaline) basalts and basaltic andesites also occur (Supplementary Fig. S1). The alkalic and tholeiitic basalts have similar Fe_2_O_3_* contents at a given MgO content (Supplementary Fig. S2). The chemistry of incompatible trace elements has an affinity to intra-plate basalt but with significant elevations in Ba, Pb, and Sr concentrations (Supplementary Fig. S3a), which are fluid mobile. The alkaline basalts have relatively high concentrations of incompatible elements compared to the tholeiitic basalts.

Water content of the Changbaishan basalts have yet to be successfully measured, because the basalts have been subaerially emplaced and so they are commonly degassed. Our preliminary analyses showed that pyroxene and plagioclase phenocrysts and melt inclusions in minerals underwent a significant loss of water, even in the quenched scoria samples, probably due to slow ascent of the magmas through thick continental lithosphere (~100 km thick^22^). In this study, water content was estimated by applying plagioclase-melt hygrometry on a young (<1 Ma) trachy-basalt scoria sample, H2505 (Supplementary Fig. S4; Supplementary Table S1). Similarly, the minimum water content of basaltic melt was also measured by analyses of melt inclusions contained in olivine phenocrysts in a basaltic trachy-andesite sample, H90713 (Supplementary Fig. S5; Supplementary Table S1). This sample was collected from the Plinian fall deposits (Unit B of Phase 2) of the 10^th^ century caldera-forming eruption^23^. This sample is better suited, because the basaltic magma was explosively ejected during the violent Plinian eruption and then quenched. Therefore, the loss of volatiles from the melt inclusions would be minimal because of the high ascent rate of the magmas. Unfortunately, no tholeiitic basalts are found in the 10^th^ century Plinian fall deposits.

Pre-eruption water content of the H2505 basalt was estimated using plagioclase–melt equilibria assuming that the plagioclase phenocryst with the highest An content [Ca/(Ca + Na) × 100] of 77.5 (Supplementary Fig. S6) was in equilibrium with the host magma. The hygrometer of ref.^24^ yields a water content of ~1.8 ± 0.4 wt.% (Supplementary Methods). Water contents of melt inclusions in olivine phenocrysts in the H90713 basaltic trachy-andesite were determined using secondary ion mass spectrometry (SIMS)^25^. The analysis showed that the water content of the basaltic melt was >~1.7 wt.% (Supplementary Methods). If the effect of the post-entrapment inner growth of the host olivine is considered, the H_2_O content of the original melt inclusion was > ~1.5 wt.% (Supplementary Methods). The calculated major element composition of the original melt inclusion is near the whole-rock composition of H2505 (Supplementary Fig. S8; Supplementary Table S1). Therefore, the H_2_O content of ~1.8 ± 0.4 wt.% estimated using plagioclase–melt hygrometry for H2505 is consistent with > ~1.5 wt.% from the melt inclusion in H90173. Based on the constraint of 1.4–2.2 wt.% obtained by the plagioclase–melt hygrometry and that of > ~1.5 wt.% by the SIMS analysis, we deem that the water content of the H2505 magma was 1.5–2.2 wt.%.

The phenocryst assemblage of H2505 is olivine and plagioclase, and no significant Eu anomaly is observed in the rare-earth-element pattern of the whole-rock sample (Supplementary Fig. S3b). Therefore, it can be considered that the H2505 magma derived from the primary magma solely by olivine fractionation. The majority of Fo content [Mg/(Mg + Fe) × 100] of olivine crystals in peridotite xenoliths from Changbaishan, which may represent newly accreted lithospheric mantle underneath the craton, is ~90^26^. Therefore, given that the primary magma was in equilibrium with the mantle olivine with Fo content of 90, the primary magmatic composition was calculated using the olivine maximum fractionation model^27^ (Supplementary Table S1; Supplementary Fig. S8). The trace element and water content of the primary magma were calculated assuming that the concentrations of these elements were negligible in the fractionated olivine. Finally, the water content of the primary alkaline basaltic magma was estimated to be 1.2–1.8 wt.%. This result can be tested further by the water contents of mineral melt inclusions in the felsic products from Changbaishan^28^. The felsic magmas evolved from a parental alkaline basaltic magma primarily by fractional crystallisation without significant crustal assimilation^23^. Given that the water content of the melt inclusions with the highest H_2_O/Zr ratio represents the least water leakage from the inclusion, the water content in a primary alkaline basaltic magma is estimated to be >1.2 wt.% using the H_2_O/Zr ratio of 0.006^28^ and a Zr content of primary basaltic magma of 207 ppm (Supplementary Table S1). This result is in good agreement with the estimate of our study.

Melting conditions of the source mantle for the Changbaishan basalts were determined by applying the Ocean Basalt Simulator version 1 model (OBS1)^20^ using the estimated trace element and water contents of the primary H2505 alkaline basaltic magma (Supplementary Table S1). The analytical results show that (1) the fraction of the pyroxenite component in the source mantle was 4–11%; (2) the degree of melting was 3–6%; (3) the water content in the source mantle was 350–550 ppm; (4) the pressure and temperature of the melt segregation were 2.6–2.8 GPa and 1340–1380 °C, respectively; and (5) the mantle potential temperature was 1314–1357 °C (Supplementary Methods). It is noteworthy that the water content of the source mantle of 350–550 ppm is significantly higher than that of a normal mid-ocean ridge basalt (N-MORB) source mantle of 120 ppm^29^. To check the aforementioned results obtained by OBS1, the melting temperature of the primary magma was independently estimated using the major element composition and the water content (Supplementary Table S1). The olivine liquidus temperature for the anhydrous primary magma was calculated using the alphaMELTS model operated in the pMELTS mode^30,31^. The calculated temperature was then corrected to obtain the liquidus temperature for the hydrous primary magma containing 1.2–1.8 wt.% H_2_O using the model provided in ref.^32^. The estimated temperature for the primary magma was 1357–1387 °C at 2.6–2.8 GPa (the pressure from OBS1). This is in good agreement with the estimated melting temperatures of 1340–1380 °C using OBS1. The mantle potential temperature was also estimated using the model of ref.^21^. The model results showed that, for water contents of the primary magma of 1.2 wt.% and 1.8 wt.%, the mantle potential temperatures were 1372 ± 32 °C and 1356 ± 25 °C, respectively.

## Discussion

Geophysical studies have suggested that the mantle plume beneath the Changbaishan volcano is derived from the MTZ^7^. However, the source of the buoyancy of the mantle plume, i.e. thermal^33^, compositional^11,18,34^, or both^7,35^, remains controversial, because the water contents of the source mantle have yet to be reliably estimated. We estimated the mantle potential temperature for the plume as 1310–1360 °C by OBS1 and 1330–1400 °C by the model of ref.^21^, considering water. These temperature ranges are essentially within the range of 1300–1400 °C for MORB^4–6^, which represents the temperatures of the normal ambient upper mantle. This observation suggests that the Changbaishan mantle plume is not significantly excessively hot compared to the temperature of the ambient mantle, and therefore, we conclude that thermal buoyancy is not a primary cause of the mantle plume at least for the youngest activity of Changbaishan which corresponds with the present-day snapshot of the seismologically detected mantle plume. We note that the magmatism of the alkaline trachy basalt, for which we estimated the water content using SIMS, was active ~1000 years ago, and therefore, the present-day mantle potential temperature is well represented by the estimate of 1310–1400 °C.

**Figure 2:**
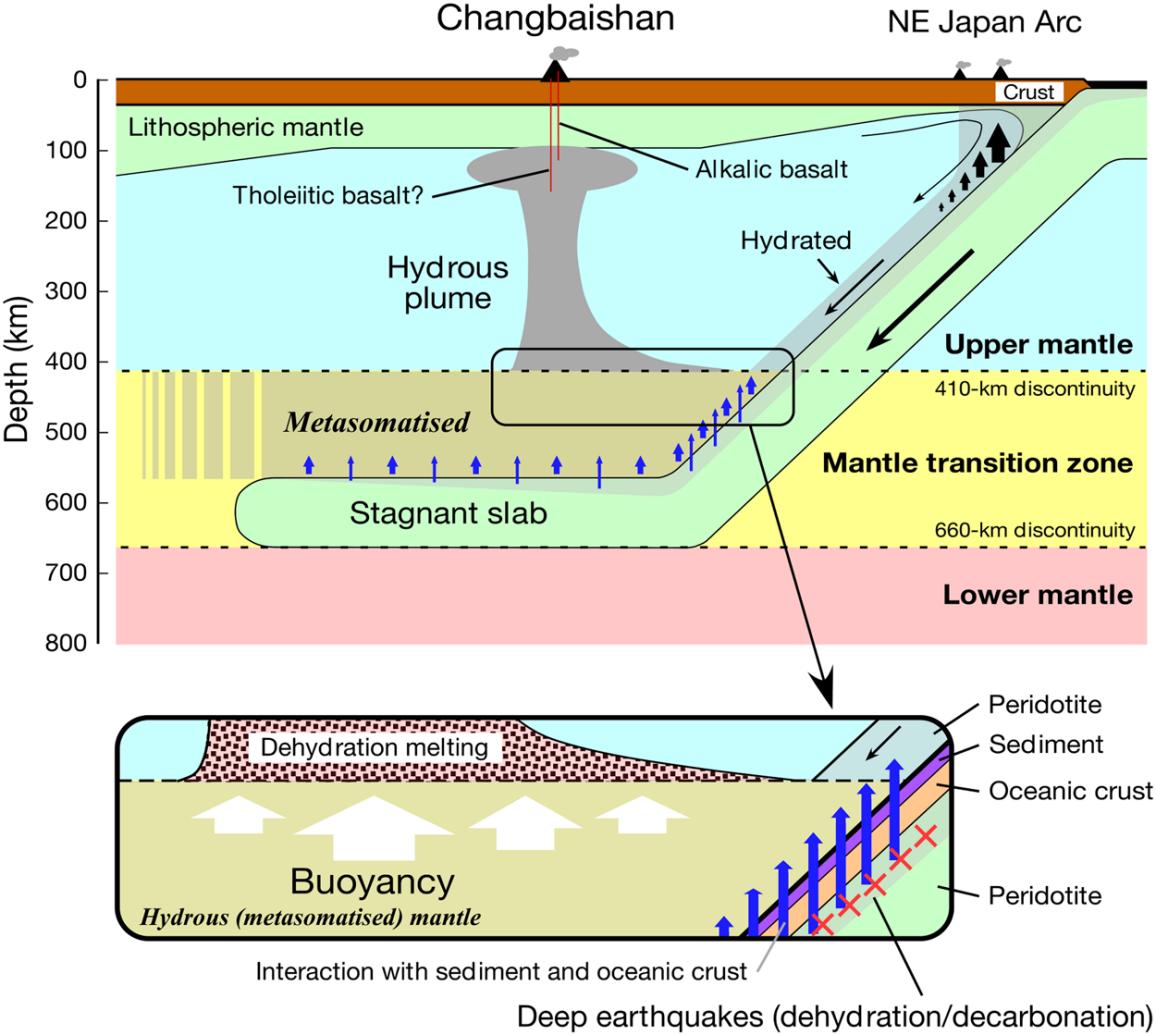
Schematic illustration of the origin of the mantle plume from the hydrous mantle transition zone under the Changbaishan intraplate volcano. See the text for details.

The source mantle of the Changbaishan basalts is considered to contain some sediment component from the subducted Pacific slab^18^. In addition, an ancient sediment component, most probably in the metasomatised mantle in the MTZ, is also associated with the source^18^ (Supplementary Fig. S9). This study and earlier works^19,22^ show the involvement of appreciable amounts of a pyroxenite component of basaltic oceanic crust in origin and H_2_O in the source mantle. It is unlikely that the basaltic oceanic crust (eclogite) and the sediments were the source of the buoyancy, because eclogite and continental crustal materials are denser than the ambient peridotite in the depth range of the deep upper mantle and MTZ^36,37^. In contrast, hydrous mantle peridotite is less dense than dry mantle peridotite, and the hydrous fluids and partial melts can also be less dense than the host hydrous peridotite in the MTZ^34^. Therefore, hydrated mantle peridotite beneath the basaltic oceanic crust in the subducted Pacific plate and/or peridotite in the MTZ above the slab are the sources of the compositional buoyancy that formed the hydrous mantle plume beneath Changbaishan (Fig. 2).

Prolonged subduction of the initially cold slab and subsequent storage in the MTZ resulted in thermal relaxation of the slab by conductive heat from the surrounding mantle, which can cause volatilisation of the slab^9,38^. Dehydration of the stagnant Pacific slab in the MTZ is suggested from deep earthquakes within the slab^39^ (Fig. 1b). The dehydration of the slab peridotite in the MTZ and/or CO_2_ release from the carbonated basaltic slab could have occurred in the MTZ and played an important role in the rehydration or re-carbonation of the peridotite in the MTZ^9,34,35^. Electrical conductivity studies have suggested that the MTZ beneath Northeast China is hydrous^16^ with as much as ~1 wt.% water^17^. A numerical study suggests that a wet peridotite plume could be generated at the top of the MTZ if ~0.5 wt.% water is contained^11^. These observations and discussions support the possibility that the Changbaishan mantle plume formed as a result of upwelling of the hydrated (and carbonated) mantle from the MTZ without significant thermal buoyancy, and as such the hydrated mantle contained chemical components from the sediments and the basaltic oceanic crust carried by the hydrous (and perhaps carbonaceous) fluids from the stagnant slab (Fig. 2).

Soon after the ascent of the plume from the MTZ to the upper mantle, dehydration melting should occur because of the reduction in water solubility in the constituent nominally anhydrous minerals^40^ (Fig. 2). A prominent low-velocity anomaly is observed at a depth range of 100–410 km beneath Changbaishan (Fig. 1b), suggesting that hydrous (and CO_2_-bearing) partial melting occurs within the entire depth range of the upwelling plume. The estimated water content in the source mantle for the H2505 alkaline basalt is only 350–550 ppm. The relatively low water content may be because the primary H2505 magma represents the melt segregated from the shallowest part of the ascending plume at ~100 km depth (i.e. 2.6–2.8 GPa).

A more hydrous magma would be generated from deeper levels of the mantle plume. The majority of the basaltic magmas are alkaline, but tholeiitic basalts also have erupted from the Changbaishan volcano (Supplementary Fig. S1). Alkaline basalts and tholeiitic basalts coexist in some Cenozoic intraplate volcanoes in Northeast China, which have been explained by two contrasting scenarios: (1) alkaline basalts formed as a result of partial melting of the asthenospheric mantle and tholeiitic basalts from partial melting of the sub-continental lithospheric mantle heated by asthenospheric melts^41^, and (2) both alkaline and tholeiitic basalts formed from the same two-lithology plume with different degrees of partial melting of the embedded pyroxenite and surrounding peridotite mantle^22^. The Pb isotopic compositions of the Changbaishan basalts are distinct from the majority composition of the sub-continental lithospheric mantle, and those of the tholeiitic and alkaline basalt compositions essentially overlap (Supplementary Fig. S9). This observation suggests that both the Changbaishan tholeiitic and alkaline basalts derived from the same mantle plume. This is consistent with the observation that the Fe_2_O_3_* contents of the tholeiitic and alkaline basalts are similar at a given MgO content^42^ (Supplementary Fig. S2). The tholeiitic basalts should have been derived from a greater degree of melting of the source mantle compared to the alkaline basalts, as has been suggested for the Northeast China intraplate basalts^22^. Onset of adiabatic melting of the tholeiitic basalt source mantle should be deeper (Fig. 2) because of either a high mantle potential temperature or high water content^22^. Determination of the water content in the tholeiitic basalts is a crucial test for the hydrous mantle plume hypothesis. However, tholeiitic basalts younger than 0.5 Ma have not been found^43^. In addition, water content could not be obtained for the tholeiitic basalts because of a lack of quenched samples suitable for water content analysis.

Concerning the origin of the Changbaishan mantle plume, one research group has developed a teleseismic tomography model beneath Northeast Asia and suggested that the stagnant slab in the MTZ has a hole beneath Changbaishan, through which hot and buoyant mantle materials ascend from the lower mantle, across the stagnant slab, and reach the upper mantle^33^. However, the teleseismic tomography method used has an inherent limitation, i.e. the background mean velocity at each depth in the study volume is removed during the computation of the teleseismic relative travel times which are used as data in the tomographic inversion. Thus, teleseismic tomography alone is unable to recover the real structure of a network-wide feature at a given depth range beneath a seismic network, such as the wide and flat stagnant slab in the MTZ beneath Northeast Asia. To resolve this problem, Chen *et al*.^44^ developed a new and robust three-dimensional tomographic model of the mantle beneath Northeast Asia by conducting a joint inversion of both local-earthquake arrival times and teleseismic relative travel times. Their model shows that there is no hole in the stagnant slab beneath Changbaishan. The results of our analyses regarding the potential temperature of the mantle plume does not suggest any advective heat flux through the stagnant slab, and thus it is consistent with the scenario that there is no hole in the stagnant slab.

Recently, Kimura *et al*.^22^ proposed that the mantle upwelling beneath Changbaishan reflects a plume that branched off from a lower mantle-derived plume beneath the Datong volcanic field in Northeast China (Fig. 1), which migrated horizontally over the stagnant slab to the east and penetrated into the upper mantle during the opening of the Sea of Japan at 30–15 Ma caused by the slab rollback of the Pacific Plate. However, the current volcanic activity at Datong is much weaker than that at Changbaishan. In addition, the present-day low-V_P_ plume structure at depths of ~300–410 km extends its trail to the east (indicated by the arrow in Fig. 1b) under which deep earthquakes in the MTZ have occurred in the Pacific slab. This observation suggests that dehydration of the stagnant slab, associated with deep earthquake faulting, is linked to the generation of the hydrous mantle plume beneath Changbaishan^39^ (Fig. 2).

Recent petrological studies have suggested that some hydrous magmas from the Siberian Traps LIP (252–250 Ma) may have occurred as a result of mantle plumes derived from the hydrous MTZ^9^, while hydrous LIP magmatism at Tarim (~290 Ma) and Emeishan (~260 Ma) is considered to have been sourced from hydrous materials from the MTZ entrained by a deep hot plume source^45^. In the ancient Earth, the mantle was hotter and significant water could not have been transported to the Earth’s deep interior via subduction of the oceanic plates because of the total breakdown of the hydrous minerals in the slab^46^ and low water solubility in the nominally anhydrous minerals in the mantle^47^. However, the mantle would have been sufficiently cool after ~1 Ga for water to be effectively transported to the MTZ^18^, and the MTZ would have become locally hydrated because of dehydration/decarbonation of the stagnant slabs^48^. The mantle plumes of the hydrous Siberian Traps magmatism could also have been generated from hydrous plumes from an intensively hydrated MTZ^9^ without significant excessive heat. Our study of the Changbaishan alkaline basalts suggests the important role of water from the stagnant slab in the MTZ as the source of the formation of the hydrous mantle plume. The roles of the hydrous mantle in the MTZ might vary and shed new light on the discussion of the origin of hydrous basalts in intra-continental basalt fields and continental LIPs.

## Methods

### Whole-rock compositional analyses

Concentrations of whole-rock major elements and some trace elements (Sc, V, Cr, Co, and Ni) were determined using X-ray fluorescence (XRF) using a Spectoris MagiX PRO at the Graduate School of Science, Hokkaido University. Additional trace elements were analysed using inductively coupled plasma mass spectrometry (ICP–MS), using a Thermo Fisher Scientific X-series instrument at Hokkaido University. Sr, Nd, and Pb isotopic ratios were determined using a multiple collector (MC)–ICP–MS (Neptune plus, Thermo Fisher Scientific) at Hokkaido University. The details of the analytical procedures, including those of the chemical separations and data correction, are described in ref.^49^. The composition of the Geological Survey of Japan reference material JB-3 was measured during the course of this study; the measured and reference values are listed in Table A1 of ref.^49^.

### Electron microprobe analyses

Compositions of minerals and glass were determined using a JEOL JXA-8800 electron microprobe at Hokkaido University. For olivine, an accelerating voltage of 15 kV; a beam current of 20 nA; peak and background counting times of 20 s and 10 s, respectively; and a focused beam were adopted. Operating conditions for plagioclase were an accelerating voltage of 15 kV; a beam current of 10 nA; peak and background counting times of 10 s and 5 s, respectively; and a beam diameter of 10 μm. Operating conditions for glass were an accelerating voltage of 15 kV; a beam current of 10 nA; peak and background counting times of 10 s and 5 s, respectively; and a beam diameter of 30–50 μm. Both oxide and natural mineral standards were used, and data were obtained using the ZAF correction method.

## Supplementary information

Supplementary Information

## Data Availability

The datasets generated and/or analysed during the current study are available from the corresponding author upon reasonable request.
