# Supplementary material for: Buoyant hydrous mantle plume from the mantle transition zone
Source: Sci Rep. 2019 Apr 25;9:6549. doi: 10.1038/s41598-019-43103-y (PMC6484022; doi:10.1038/s41598-019-43103-y)
Supplement: Supplementary file 1 — Supplementary Information [file 41598_2019_43103_MOESM1_ESM.pdf]

Supplementary Information

Article in *Scientific Reports*

Buoyant hydrous mantle plume from the mantle transition zone

Takeshi Kuritani[1]*, Qun-Ke Xia[2], Jun-Ichi Kimura[3], Jia Liu[4], Kenji Shimizu[5], Takayuki Ushikubo[5], Dapeng Zhao[6], Mitsuhiro Nakagawa[1], Shumpei Yoshimura[1]

[1]Graduate School of Science, Hokkaido University, Sapporo, Japan

[2]School of Earth Sciences, Zhejiang University, Hangzhou, China

[3]Department of Solid Earth Geochemistry, Japan Agency for Marine-Earth Science and Technology, Yokosuka, Japan

[4]Key Laboratory of Submarine Geosciences, Second Institute of Oceanography, State Oceanic Administration, Hangzhou, China

[5]Kochi Institute for Core Sample Research, Japan Agency for Marine-Earth Science and Technology, Nankoku, Japan

[6]Graduate School of Science, Tohoku University, Sendai, Japan

*Corresponding author: kuritani@sci.hokudai.ac.jp

Supplementary Figures

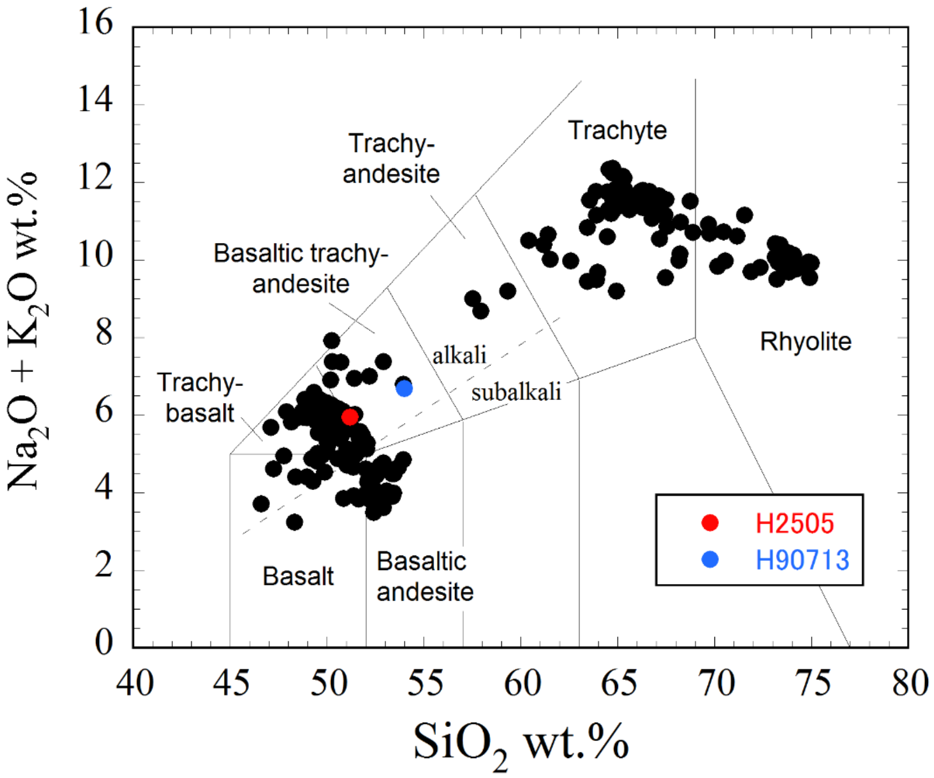

Supplementary Figure S1. Major element compositions of eruptive products from the Changbaishan volcano, shown in a total alkali–SiO_2 diagram. Data are from this study and refs.[1–9].

(a)

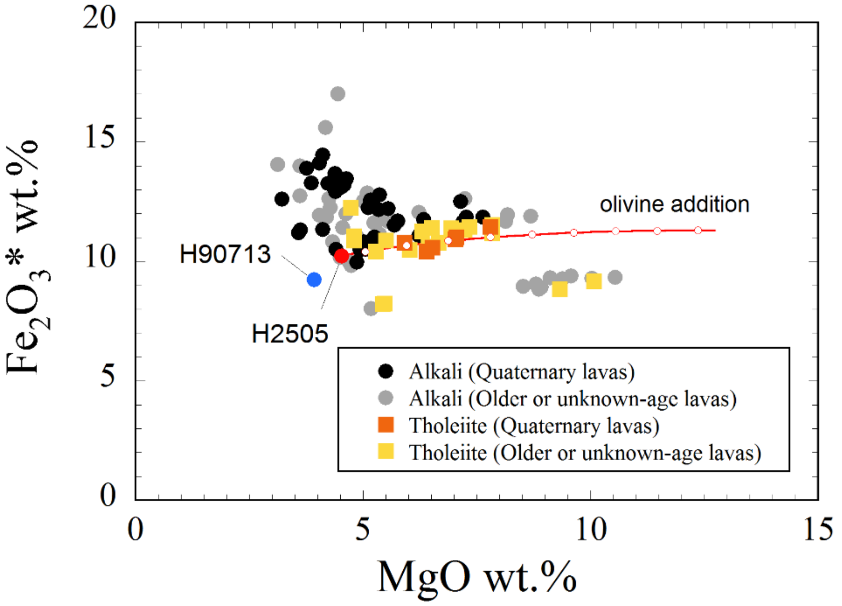

(b)

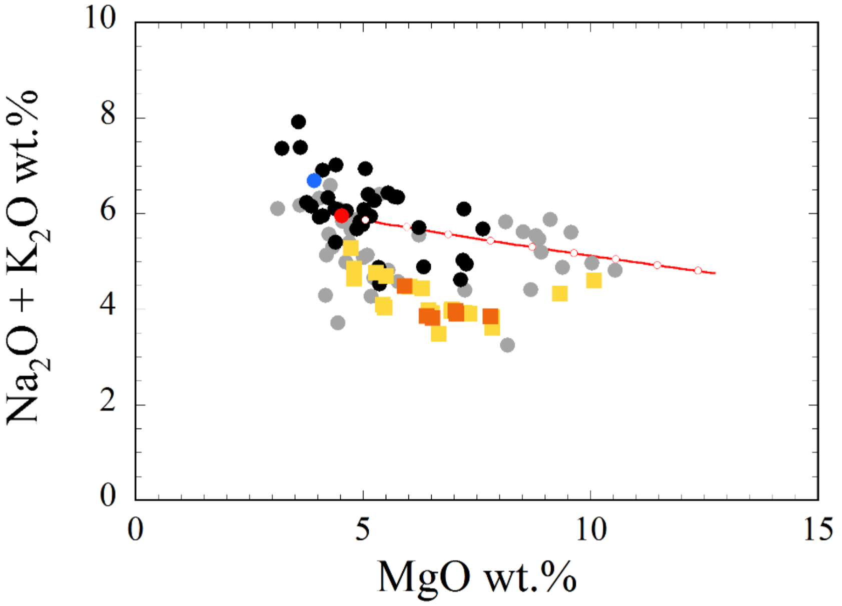

Supplementary Figure S2. The whole-rock compositions of mafic products from the Changbaishan volcano, shown in (a) Fe_2O_3*–MgO and (b) total alkali–MgO diagrams. The trajectories from the composition of H2505 indicate the paths predicted by the olivine maximum fractionation model. Data are from this study and refs.[1,2,5,7,8].

(a)

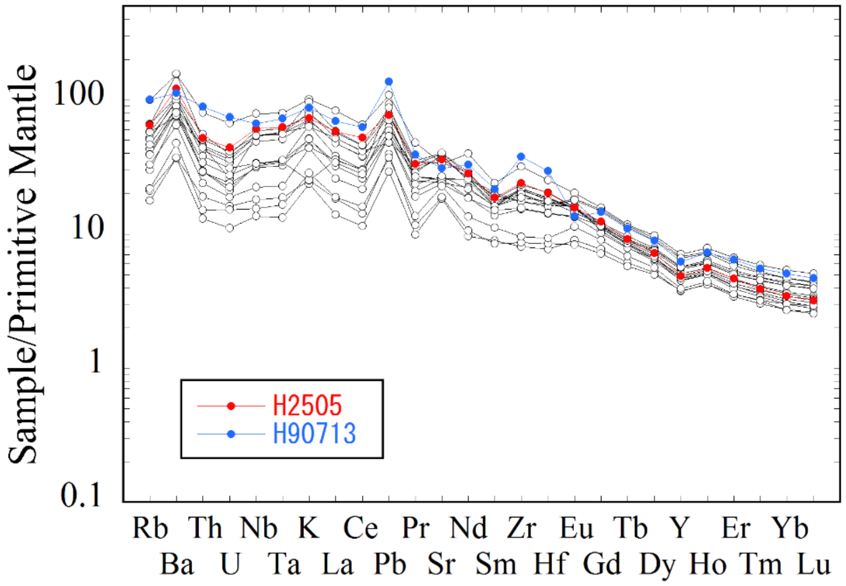

(b)

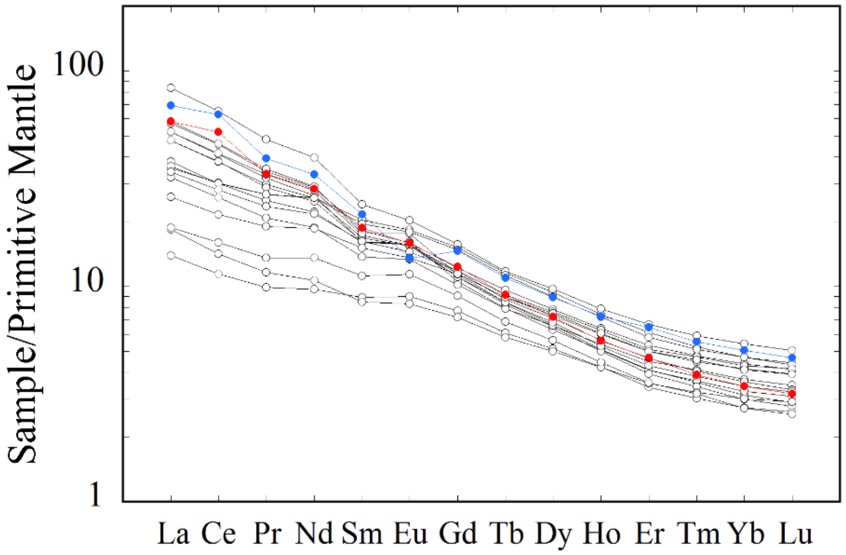

Supplementary Figure S3. Primitive mantle-normalized (a) multi-element concentration diagram and (b) REE concentration diagram for mafic products from the Changbaishan volcano. Data are from this study and ref.[8]. The trace element concentrations of primitive mantle are from ref.[10].

(a)

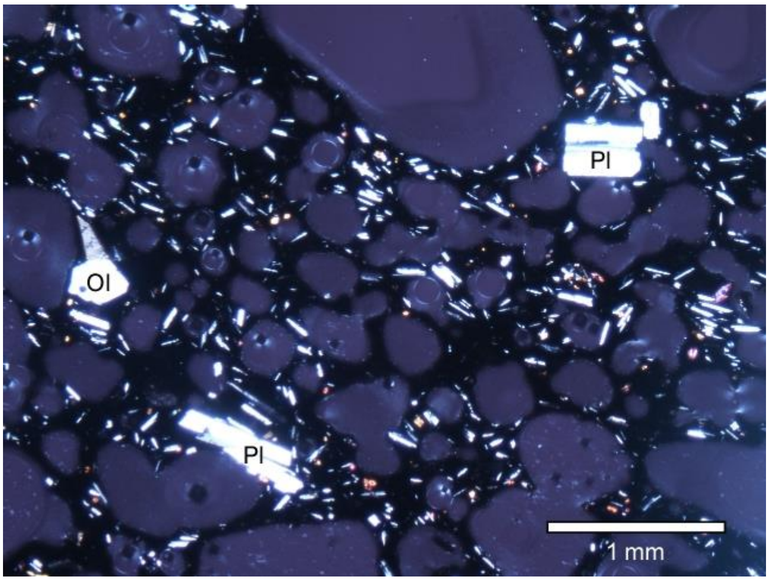

(b)

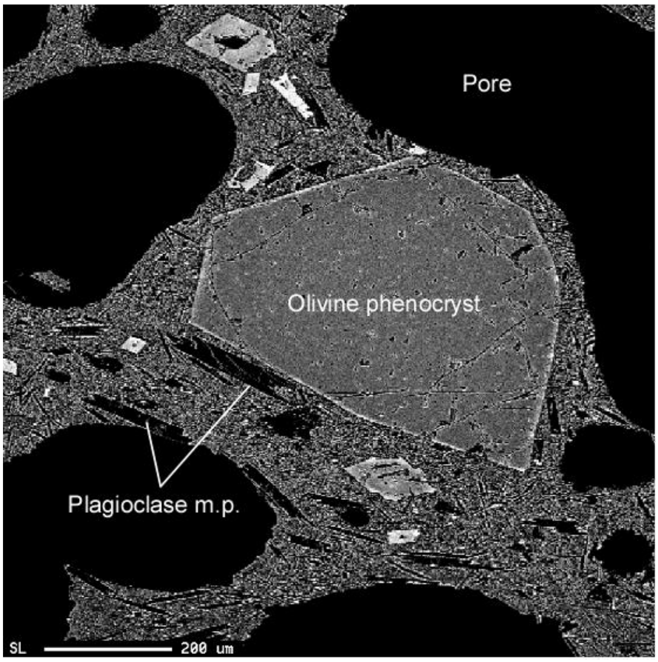

Supplementary Figure S4. (a) Photomicrograph (crossed-polarized light) and (b) back-scattered image of the sample H2505. “m.p.” denotes microphenocryst.

(a)

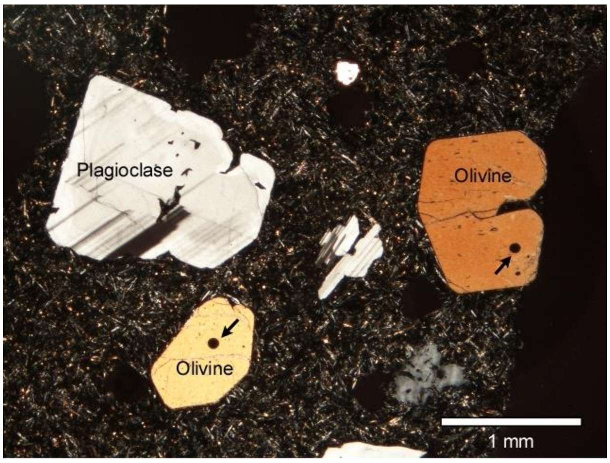

(b)

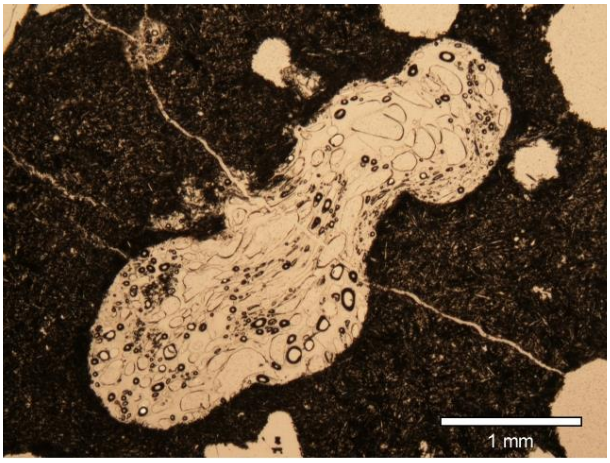

(c)

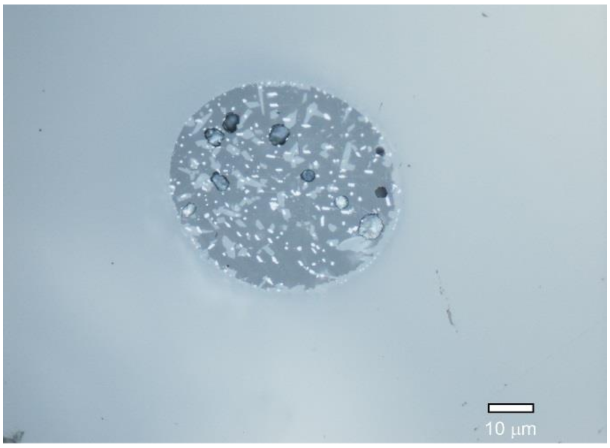

Supplementary Figure S5. Photomicrographs of the sample H90713, showing (a) olivine and plagioclase phenocrysts (crossed-polarized light), (b) blobs of felsic glass (parallel-polarized light), and (c) a melt inclusion (reflected light). Arrows in (a) indicate melt inclusions in olivine phenocrysts.

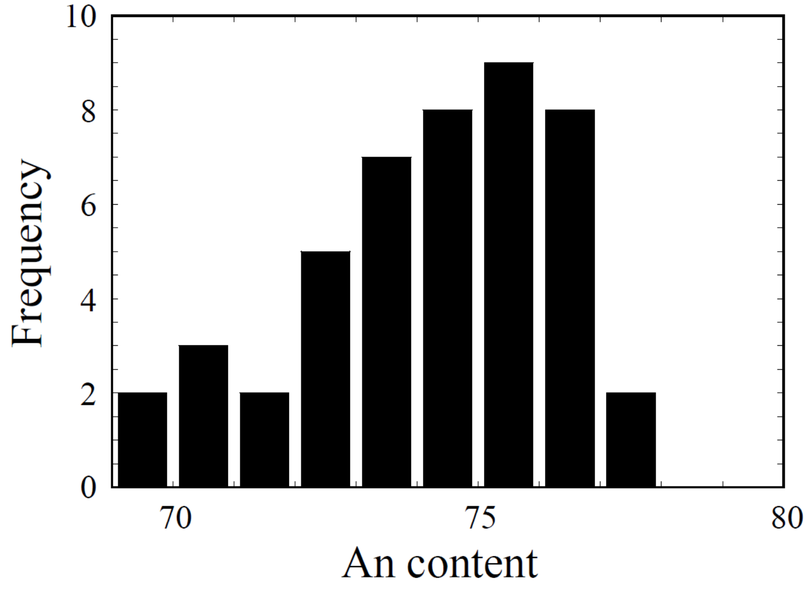

Supplementary Figure S6. Histogram of the An content of the cores of plagioclase phenocrysts in the sample H2505.

(a)

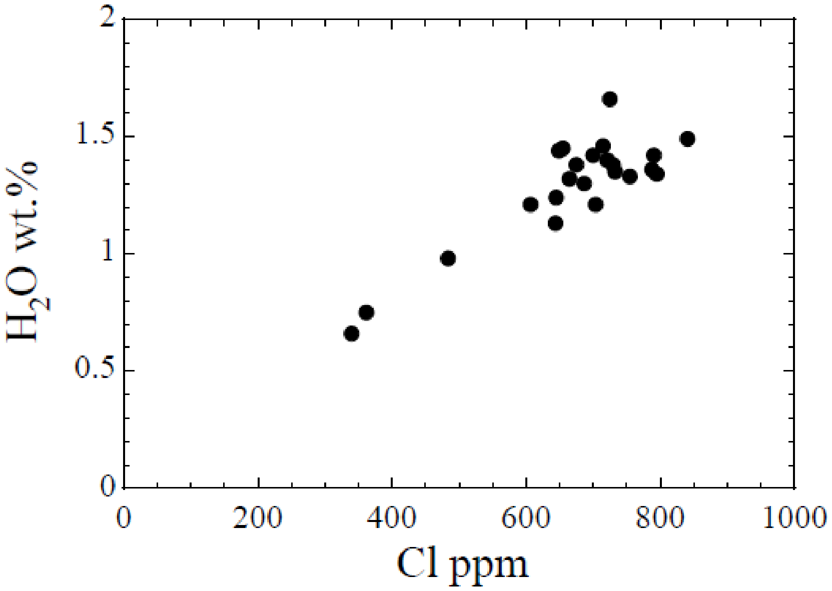

(b)

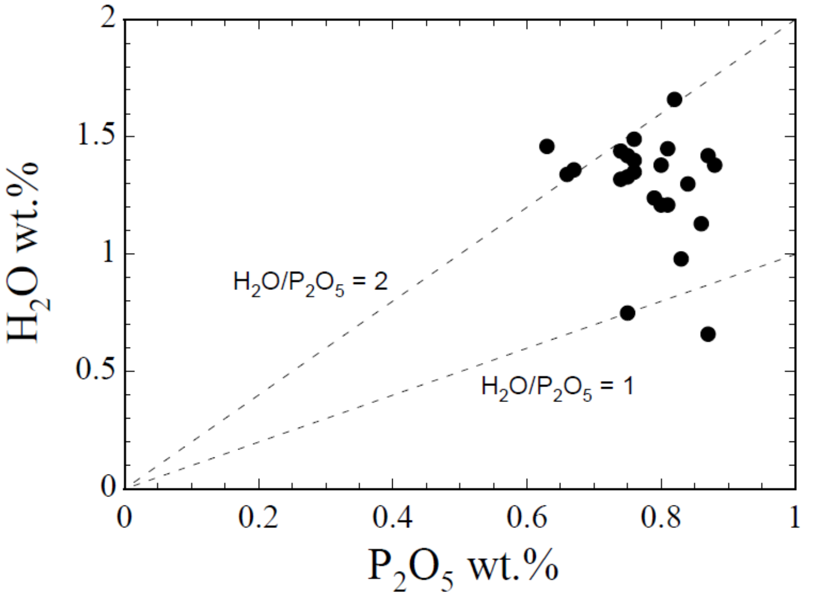

Supplementary Figure S7. H_2O contents of melt inclusions in olivine phenocrysts in H90173, against (a) Cl and (b) P_2O_5 contents.

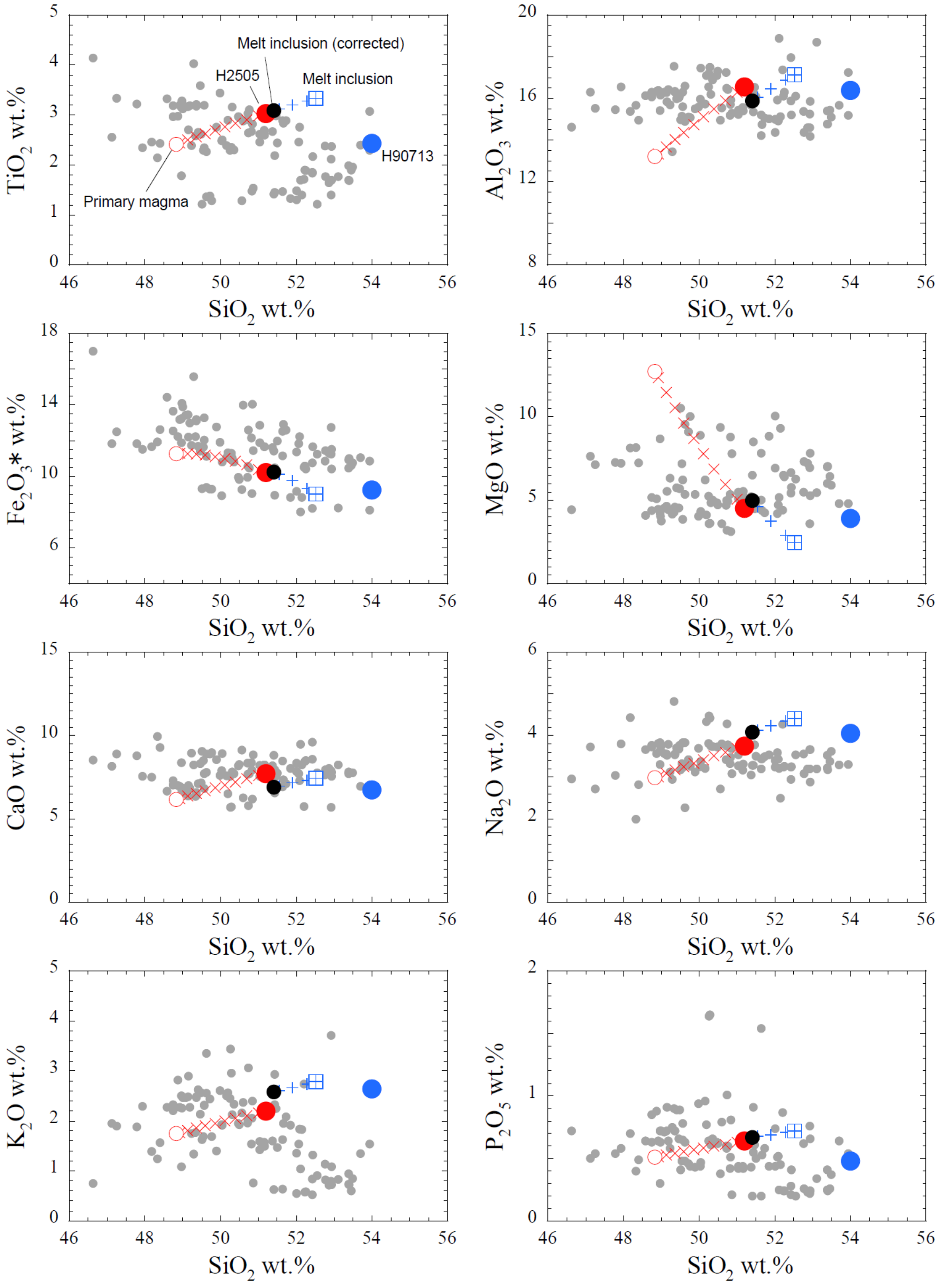

Supplementary Figure S8. The whole-rock compositions of mafic products from the Changbaishan volcano, along with the composition of the melt inclusion and the corrected composition of the melt inclusion. The estimated primary magma composition for H2505 is also shown. The trajectories from the compositions of the melt inclusion and H2505 indicate the paths predicted by the olivine maximum fractionation model.

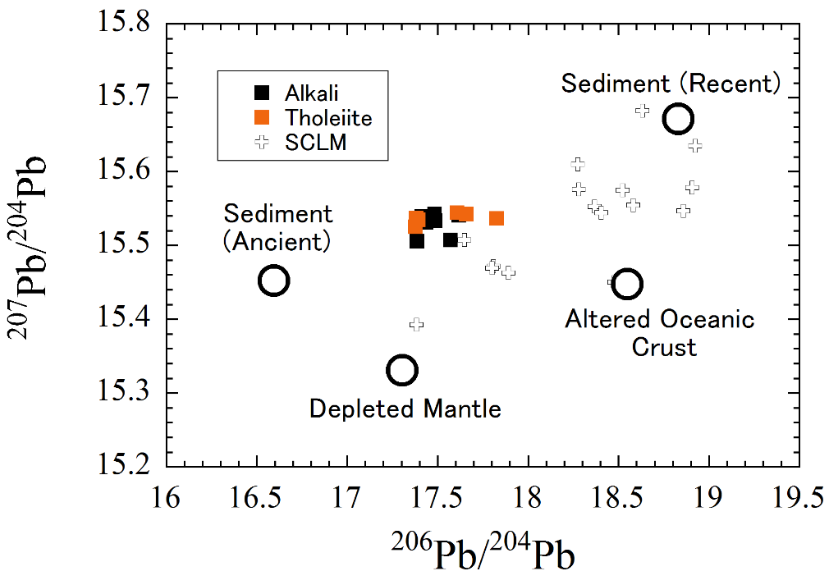

Supplementary Figure S9. The Pb isotopic ratios of the mafic products from the Changbaishan volcano. Data are from this study and ref.[8]. The SCLM (sub-continental lithospheric mantle) data are from mantle xenoliths from Korea[11]. Large open circles show the present-day compositions of the possible mantle components (recent sediments, ancient sediments, altered oceanic crust, and depleted mantle) for the Changbaishan magmas. The compositions of the sediments and depleted mantle are from ref.[12] and that of the altered oceanic crust is from ref.[13].

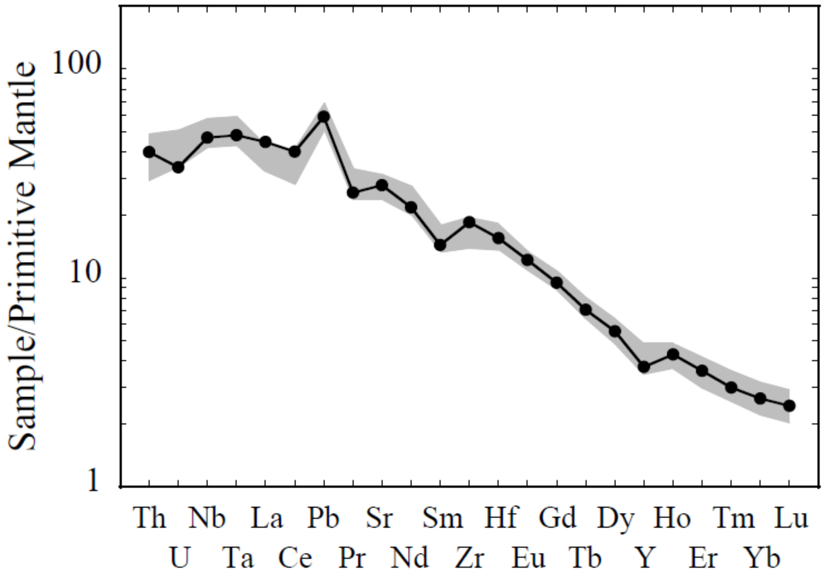

Supplementary Figure S10. Comparison of the composition of the estimated primary H2505 magma (filled circles) with those predicted by the OBS1 model (area in grey) in a primitive mantle-normalized multi-element concentration diagram. The trace element concentrations of primitive mantle are from ref.[10].

Supplementary Tables

Supplementary Table S1: Whole-rock compositions of the studied samples

Sample	H90713			H2505	
	Whole rock	Melt inclusion[#]	Melt inclusion (corrected)	Whole rock	Primary magma
Major elements (wt.%)					
SiO_2	53.69	52.52	51.40	51.56	48.74
TiO_2	2.41	3.33	3.09	3.05	2.39
Al_2O_3	16.29	17.15	15.89	16.68	13.08
Fe_2O_3*	9.19	9.03	10.26	10.31	11.29
MnO	0.13	0.13	0.12	0.13	0.10
MgO	3.91	2.46	4.98	4.57	13.09
CaO	6.70	7.45	6.91	7.78	6.10
Na_2O	4.03	4.41	4.09	3.78	2.96
K_2O	2.62	2.79	2.58	2.21	1.74
P_2O_5	0.47	0.72	0.67	0.64	0.51
Total	99.46	100.00	100.00	100.71	100.00
LOI	1.63	-	-	0.25	-
H_2O	-	>1.66	>1.54	1.5-2.2	1.2-1.8
Mg#	45.7	35.0	49.0	46.7	69.7
Trace elements (ppm)					
Sc (XRF)	15	-	-	19	-
V (XRF)	126	-	-	174	-
Cr (XRF)	71	-	-	64	-
Co (XRF)	30	-	-	33	-
Ni (XRF)	43	-	-	40	-
Rb	63.9	-	-	41.2	31.7
Sr	656	-	-	760	585
Y	28.4	-	-	22.1	17.0
Zr	422	-	-	269	207
Nb	47.6	-	-	43.5	33.5
Cs	0.83	-	-	0.35	0.27
Ba	784	-	-	846	652
La	47.6	-	-	40.0	30.8
Ce	111	-	-	92.7	71.4
Pr	10.8	-	-	9.18	7.07
Nd	44.7	-	-	38.3	29.5
Sm	9.58	-	-	8.30	6.39
Eu	2.27	-	-	2.66	2.05
Gd	8.72	-	-	7.35	5.66
Tb	1.18	-	-	0.99	0.76
Dy	6.55	-	-	5.31	4.09
Ho	1.19	-	-	0.92	0.70
Er	3.10	-	-	2.24	1.72
Tm	0.41	-	-	0.29	0.22
Yb	2.49	-	-	1.69	1.30
Lu	0.35	-	-	0.23	0.18
Hf	9.07	-	-	6.23	4.80
Ta	2.97	-	-	2.57	1.98
Pb	9.75	-	-	5.45	4.20
Th	7.57	-	-	4.42	3.40
U	1.57	-	-	0.92	0.71
Isotopes					
$^{87}Sr/^{86}Sr$	0.704918±8	-	-	0.704941±25	-
$^{143}Nd/^{144}Nd$	0.512596±5	-	-	0.512616±5	-
$^{206}Pb/^{204}Pb$	17.5096±4	-	-	17.4831±5	-
$^{207}Pb/^{204}Pb$	15.5420±5	-	-	15.5340±5	-
$^{208}Pb/^{204}Pb$	37.8991±9	-	-	37.8983±12	-

Fe_2O_3*: total Fe as Fe_2O_3.
Errors on isotope ratios are within-run 2SE.
#: The average of the EPMA data.

Supplementary Table S2: Compositions of the representative olivine and plagioclase phenocrysts in H2505 and H90713

Sample	H2505						H90713		
	Olivine			Plagioclase			Olivine		
	#1	#16	#33	#L3	#L7	#62	#8	#13	#22
SiO_2	39.14	39.25	39.46	49.71	49.91	49.92	38.92	39.64	38.74
Al_2O_3	0.03	0.03	0.02	31.91	31.89	31.38	0.04	0.03	0.03
FeO*	20.58	20.24	19.44	0.48	0.50	0.53	20.70	19.26	21.31
MnO	0.22	0.26	0.23	-	-	-	0.29	0.23	0.26
MgO	40.03	40.24	41.21	0.15	0.10	0.10	40.01	41.30	39.53
CaO	0.21	0.24	0.20	15.48	15.44	15.22	0.25	0.21	0.24
Na_2O	-	-	-	2.55	2.48	2.53	-	-	-
K_2O	-	-	-	0.23	0.17	0.19	-	-	-
NiO	0.20	0.07	0.13	-	-	-	0.12	0.14	0.13
Total	100.41	100.32	100.69	100.51	100.50	99.86	100.33	100.83	100.26
Fo or An	77.6	78.0	79.1	77.0	77.5	76.9	77.5	79.3	76.8

FeO*: total Fe as FeO.

Supplementary Table S3: Compositions of representative olivine-hosted melt inclusions in H90713 and that of groundmass glass in H2505

Sample	H90713									H2505
	Melt inclusion									Groundmass glass#
	#1	#2	#3	#4	#5	#6	#7	#8	#9	
SiO_2	51.31	52.95	52.78	52.82	51.88	51.14	50.89	51.00	50.68	50.67
TiO_2	3.29	3.30	3.00	3.77	3.63	2.91	2.98	3.46	3.29	3.68
Al_2O_3	16.71	17.47	17.04	17.36	16.94	16.22	16.68	17.11	16.38	15.79
FeO*	8.76	8.47	9.27	9.09	9.28	9.57	8.27	9.08	8.43	10.39
MnO	0.15	0.14	0.07	0.13	0.07	0.09	0.13	0.17	0.15	0.15
MgO	2.42	2.25	2.01	1.56	2.40	3.27	2.17	2.34	1.99	3.71
CaO	7.64	8.12	6.56	5.19	7.73	6.35	7.20	7.00	7.06	8.00
Na_2O	4.45	3.98	4.45	4.62	4.45	4.29	4.29	4.37	4.41	4.21
K_2O	2.49	2.62	3.35	3.53	2.71	2.70	2.83	2.86	2.87	2.47
P_2O_5	0.57	0.65	0.78	0.86	0.77	0.79	0.70	0.67	0.80	0.76
Total	97.78	99.96	99.31	98.93	99.87	97.33	96.12	98.07	96.05	99.83
Mg#	33.0	32.1	27.8	23.4	31.5	37.9	31.8	31.4	29.6	38.9

FeO*: total Fe as FeO.

#: The glass composition is the average of 80 analyses.

Supplementary Table S4: Results of volatile content analyses on melt inclusions

	CO_2 ppm	H_2O wt.%	F ppm	P_2O_5 wt.%	Cl ppm
Ol#36	23	1.49	1,235	0.76	841
Ol#37	22	1.40	1,000	0.76	721
Ol#37-2	24	1.42	1,039	0.75	700
Ol#37-4	31	1.66	1,655	0.82	725
Ol#38	20	1.46	1,101	0.63	715
Ol#40	22	1.44	937	0.74	649
Ol#40-4	48	1.32	1,065	0.74	665
Ol#42	24	1.13	949	0.86	644
Ol#42-2	-	1.42	992	0.87	791
Ol#43-1	22	1.45	1,152	0.81	655
Ol#43-2	-	0.75	694	0.75	362
Ol#45	27	1.38	1,001	0.80	675
Ol#46	32	1.30	1,300	0.84	687
Ol#47	13	0.66	797	0.87	340
Ol#48	58	1.21	1,291	0.80	607
Ol#49	-	1.35	1,229	0.76	733
Ol#49-2	22	1.24	1,224	0.79	645
Ol#50	-	1.38	1,167	0.88	730
Ol#51	22	1.36	1,337	0.67	788
Ol#51-2	23	0.98	848	0.83	484
Ol#53-1	37	1.33	1,085	0.75	755
Ol#53-2	-	1.21	961	0.81	704
Ol#54	26	1.34	1,356	0.66	795

Supplementary Methods

Water content analyses using a plagioclase–melt hygrometry

The plagioclase–melt hygrometer of ref.[14] was applied to a scoria sample (H2505; Supplementary Fig. S4). This sample is identical to the sample cha-19 of ref.[7]. We selected this sample, because it is aphyric (<3 vol.% crystals), the groundmass is glassy, and it does not show any evidence of magma mixing. The sample contains olivine and plagioclase phenocrysts. The Fo content of the olivine phenocrysts ranges from 77 to 79 and the An content of the plagioclase phenocrysts ranges from 69 to 78 (Supplementary Fig. S6). The compositions of the representative olivine and plagioclase phenocrysts are listed in Supplementary Table S2. The plagioclase phenocrysts show euhedral morphology and the europium anomaly is near unity (Supplementary Fig. S3b), suggesting that the plagioclase phenocrysts grew *in situ* in the magma. Therefore, it can be assumed that the plagioclase with the highest An content of 77.5 was in equilibrium with a melt with the whole-rock composition of H2505.

To apply the hygrometer, the temperature condition must be provided. In the sample, plagioclase microphenocrysts (<0.2 mm) commonly have swallow tails (Supplementary Fig. S4b), suggesting that they rapidly formed during the eruption. During the ascent of the water-bearing magma, water saturation occurred in the melt at depth. Large undercooling caused by the decompression of the water-saturated magma induced crystallisation because of the increase in the liquidus temperature owing to vapor exsolution[15,16]. The eruption temperature of the magma was estimated using the constraint that the melt was saturated with plagioclase. The melt composition, obtained using an electron probe micro-analyser (EPMA) on the groundmass glass, is shown in Supplementary Table S3. The water content of the glass was obtained as ~0.3 wt.% using a fourier-transform infrared spectrometer located at Hokkaido University[17]. The alphaMELTS model in the MELTS mode[18–20] showed that the magma eruption temperature was 1060ºC.

Although the pressure of the crystallisation cannot be constrained, the hygrometer of ref.[14] is not sensitive to pressure. The hygrometer yielded a water content of 1.81 wt.% and 1.75 wt.% at assumed pressure conditions of 2 kbar and 5 kbar, respectively. The temperatures were calculated using an estimate of ~1060ºC at ~20 bar and an adiabatic temperature gradient of 25.8ºC/GPa for the magma, obtained using the alphaMELTS model. The typical uncertainty of this hygrometer is 0.35 wt.% H_2O[14]. The An–Ab exchange coefficient (K_D (An–Ab)$^{plagioclase\text{-}liquid}$) increases with temperature, and the $K_D = 0.10 \pm 0.05$ at T < 1050ºC and $K_D = 0.27 \pm 0.11$ at T ≥ 1050ºC[21]. Although

the K_D of 0.13 for the plagioclase–melt pair at ~1060ºC is out of the range of K_D = 0.27 ± 0.11 at T ≥ 1050ºC, it is well within the range of K_D = 0.10 ± 0.05 at T < 1050ºC. Because the temperature condition of ~1060ºC is close to the boundary temperature of 1050ºC, we believe that the plagioclase–melt pair was close to be in equilibrium.

Water content analyses of melt inclusions.

The water contents were determined for melt inclusions in the olivine phenocrysts in a basaltic trachy-andesite sample (H90713; Supplementary Fig. S5), which was explosively ejected during the Plinian eruption of felsic magmas during the 10th century. This sample is identical to the sample 907-1-3 of ref.[9]. The sample contains ~15 vol.% plagioclase phenocrysts, ~5 vol.% olivine phenocrysts, and rare sanidine phenocrysts. Blobs of vesiculated felsic glass are observed (Supplementary Fig. S5b), suggesting that the sample was not a wall rock which was entrained by felsic magmas during eruption, but was produced by magmatic mixing. Therefore, it is expected that the melt inclusions preserve the original volatile contents at depths just prior to eruption.

The melt inclusions contain tiny (<~5 μm) daughter crystals of olivine and sulfide, in addition to glass (Supplementary Fig. S5c). The compositions of the melt inclusions, measured using EPMA with a 50-μm-diameter broad beam, are listed in Supplementary Table S3; the average composition is shown in Supplementary Table S1. The Fo contents of the host olivine are 77–79 (Supplementary Table S2). The H_2O, CO_2, F, Cl, and P_2O_5 content analyses of the melt inclusions were conducted using an ion microprobe CAMECA IMS-1280HR, AMETEK CAMECA, at the Kochi Institute for Core Sample Research, JAMSTEC. To prevent a diffusive loss of water and because of the low content of the daughter crystals (<10%), we did not homogenise the inclusions at high temperatures, and the analyses were conducted on the glass-rich domains in the inclusions. The details of the analytical procedures, including sample preparation, analytical conditions, and standard data, are provided in ref.[22]. The analytical results are listed in Supplementary Table S4. The H_2O contents are variable, and they show a good correlation with the Cl contents (Supplementary Fig. S7a), suggesting that diffusive loss of H_2O from the melt inclusions to the host olivine was not significant. In contrast, the H_2O contents do not show a positive correlation with the P_2O_5 contents (Supplementary Fig. S7b). These observations suggest that the variation in the H_2O contents was produced not by crystal fractionation but by leakage of H_2O (and Cl) possibly through cracks in the host olivine. In this case, it can be considered that the melt inclusion with the highest H_2O/P_2O_5 ratio of 2.3 was the least affected by the H_2O loss. Because the daughter olivine and sulfide crystals do not contain appreciable amounts of H_2O and

P_2O_5, the H_2O content of the melt inclusions can be estimated as ~1.7 wt.% using the P_2O_5 content of the inclusions of 0.72 wt.% (Supplementary Table S1) and the H_2O/P_2O_5 ratio of 2.3. The CO_2 contents of the inclusions are as low as <100 ppm, suggesting that CO_2-rich C–H–O gas had already been degassed from the magma at depths. Therefore, the original water contents of the inclusions were greater than ~1.7 wt.%.

The melt inclusions are considered to have undergone post-entrapment growth of the host olivine, as suggested from their low MgO contents (Supplementary Table S1). To estimate the original composition, the equilibrium olivine was incrementally added to the melt until it could coexist with the host olivine (Fo79), assuming an $Fe^{3+}/\Sigma Fe$ ratio of 0.15 and an equilibrium $(Fe^{2+}/Mg)^{olivine/melt}$ distribution coefficient of 0.3 according to ref.[23]. The corrected composition of the melt inclusion (Supplementary Table S1) is similar to the whole-rock composition of the aphyric H2505 magma (Supplementary Fig. S8). The mostly identical Fe_2O_3*contents also suggest that "Fe-loss"[24] did not significantly occur in the inclusions. The H_2O content of the corrected melt inclusion is estimated to be >~1.5 wt.%.

Estimation of mantle melting parameters

The mantle melting parameters, such as the source lithology, depth of melting, melting temperature, degree of melting, and source water content, were estimated using the Ocean Basalt Simulator version 1 model (OBS1)[25]. OBS1 is a thermodynamic-based trace element mass balance model. It allows one to estimate the melting parameters for a pyroxenite-bearing peridotite undergoing adiabatic melting, using the trace element contents and the water content of the primary magmas as input variables. The model was applied to the primary H2502 magma (Supplementary Table S1). The melting parameters were optimised such that the calculated primary compositions best match the target compositions using Monte Carlo simulation. Although the model considers a pyroxenite-bearing source mantle, it does not consider a sediment component. Because the source mantle for the Changbaishan magma contains sediments that were derived from the stagnant slab[12], the concentrations of Rb, Ba, and K, which are considered to have largely originated from the sediment component[12], were not used for the fitting.

A comparison of the target magmatic composition to the predicted magmatic composition is shown in Supplementary Fig. S10. The model results showed that, for a water content of the primary magma of 1.8 wt.%; the pyroxenite fraction in the source mantle is 6 ± 2%; the temperature and pressure at which the melt segregated are 1354 ± 15ºC and 2.7 ± 0.1 GPa, respectively; the mean degree of melting is 4.1 ± 1.0 wt.%; and

the source water content is ~550 ppm, with the melting model of “metasomatism,” a pyroxenite source of “Py-MORB,” a PERID_2 model of “DM,” and a prior melt extraction of PERID_1 of 0%. The model also yields a mantle potential temperature of 1330 ± 16ºC. For a water content of the primary magma of 1.2 wt.%, the pyroxenite fraction in the source mantle is 8 ± 3%; the temperature and pressure at which the melt segregated is 1372 ± 8ºC and 2.7 ± 0.1 GPa, respectively; the mean degree of melting is 4.9 ± 1.0 wt.%; the source water content is ~350 ppm; and the mantle potential temperature is 1350 ± 7ºC.

Supplementary references

1. Basu, A. R., Junwen, W., Wankang, H., Guanghong, X. & Tatsumoto, M. Major element, REE, and Pb, Nd and Sr isotopic geochemistry of Cenozoic volcanic rocks of eastern China: implications for their origin from suboceanic-type mantle reservoirs. *Earth Planet. Sci. Lett.* **105,** 149–169 (1991).
2. Hsu, C.-N., Chen, J.-C. & Ho, K.-S. Geochemistry of Cenozoic volcanic rocks from Kirin Province, northeast China. *Geochem. J.* **34,** 33–58 (2000).
3. Miyamoto, T., Nakagawa, M., Tanaka, Y. & Yoshida, M. Eruptive sequence of the 10th century Baitoushan eruption. *Center for Northeast Asian Studies Monograph Series* **16,** 15–43 (2004).
4. Nakagawa, M., Miyamoto, T., Tanaka, Y. & Yoshida, M. The finding and its significance of 9th century Baitoushan eruption. *Center for Northeast Asian Studies Monograph Series* **16,** 45–54 (2004).
5. Chen, Y., Zhang, Y., Graham, D., Su, S. & Deng, J. Geochemistry of Cenozoic basalts and mantle xenoliths in northeast China. *Lithos* **96,** 108–126 (2007).
6. Zou, H., Fan, Q. & Yao, Y. U–Th systematics of dispersed young volcanoes in NE China: asthenosphere upwelling caused by pilling up and upward thickening of stagnant Pacific slab. *Chem. Geol.* **255,** 134–142 (2008).
7. Hashimoto, C. Petrological study of Cenozoic volcanic rocks in Changbaishan area, northeast China –basaltic magma generation at “Big Mantle Wedge” –. Master thesis, Graduate School of Science, Hokkaido University, 139p (2010).
8. Kuritani, T., Kimura, J.-I., Miyamoto, T., Wei, H., Shimano, T., Maeno, F., Jin, X. & Taniguchi, H. Intraplate magmatism related to deceleration of upwelling asthenospheric mantle: implications from the Changbaishan shield basalts, northeast China. *Lithos* **112,** 247–258 (2009).
9. Nishimoto, J., Nakagawa, M., Miyamoto, T. & Taniguchi, H. Magma system of 10th century eruption of Baitoushan volcano: inferred from petrological and geochemical characteristics. *Center for Northeast Asian Studies Monograph Series* **41,** 71–94 (2009).
10. Sun, S.-S. & McDonough, W. F. Chemical and isotopic systematics of oceanic basalts: implications for mantle composition and processes. In: Saunders, A.D., Norry, M. J. (Eds.), Magmatism in the Ocean Basins. *Geol. Soc. London, Spec. Pub.* **42,** 313–345 (1989).
11. Choi, S. H., Kwon, S.-T., Mukasa, S. B. & Sagong, H. Sr–Nd–Pb isotope and trace element systematics of mantle xenoliths from Late Cenozoic alkaline lavas,

South Korea. *Chem. Geol.* **221,** 40–64 (2005).
12. Kuritani, T., Ohtani, E. & Kimura, J.-I. Intensive hydration of the mantle transition zone beneath China caused by ancient slab stagnation. *Nature Geosci.* **4,** 713–716 (2011).
13. Kimura, J.-I. & Nakajima, J. Behaviour of subducted water and its role in magma genesis in the NE Japan arc: a combined geophysical and geochemical approach. *Geochim. Cosmochim. Acta* **143,** 165–188 (2014).
14. Waters, L. E. & Lange, R. A. An updated calibration of the plagioclase-liquid hygrometer-thermometer applicable to basalts through rhyolites. *Am. Mineral.* **100,** 2171–2184 (2015).
15. Sisson, T. W. & Grove, T. L. Temperature and H_2O contents of low-MgO high-alumina basalts. *Contrib. Mineral. Petrol.* **113,** 167–184 (1993).
16. Wagner, T. P., Donnelly-Nolan, J. M. & Grove, T. L. Evidence of hydrous differentiation and crystal accumulation in the low-MgO, high-Al_2O_3 Lake basalt from Medicine Lake volcano, California. *Contrib. Mineral. Petrol.* **121,** 201–216 (1995).
17. Yoshimura, S., Kuritani, T., Matsumoto, A. & Nakagawa, M. Fingerprint of silicic magma degassing visualised through chlorine microscopy. *Sci. Rep.* **9,** doi:10.1038/s41598-018-37374-0 (2019).
18. Asimow, P. D. & Ghiorso, M. S. Algorithmic modifications extending MELTS to calculate subsolidus phase relations. *Am. Mineral.* **83,** 1127–1132 (1998).
19. Ghiorso, M. S. & Sack, R. O. Chemical mass transfer in magmatic processes IV. A revised and internally consistent thermodynamic model for the interpolation and extrapolation of liquid–solid equilibria in magmatic systems at elevated temperatures and pressures. *Contrib. Mineral. Petrol.* **119,** 197–212 (1995).
20. Smith, P. M. & Asimow, P. D. Adiabat_1ph: a new public front-end to the MELTS, pMELTS, and pHMELTS models. *Geochem. Geophys. Geosyst.* **6,** doi:10.1029/2004GC000816 (2005).
21. Putirka, K. D. Thermometers and barometers for volcanic systems. *Rev. Mineral. Geochem.* **69,** 61–120 (2008).
22. Shimizu, K., Ushikubo, T., Hamada, M., Itoh, S., Higashi, Y., Takahashi, E. & Ito, M. H_2O, CO_2, F, S, Cl, and P_2O_5 analyses of silicate glasses using SIMS: report of volatile standard glasses. *Geochem. J.* **51,** 299–313 (2017).
23. Roeder, P. L. & Emslie, R. F. Olivine–liquid equilibrium. *Contrib. Mineral. Petrol.* **29,** 275–289 (1970).
24. Danyushevsky, L. V., Della-Pasqua, F. N. & Sokolov, S. Re-equilibration of melt

inclusions trapped by magnesian olivine phenocrysts from subduction-related magmas: petrological implications. *Contrib. Mineral. Petrol.* **138,** 68–83 (2000).

25. Kimura, J.-I. & Kawabata, H. Ocean Basalt Simulator version 1 (OBS1): trace element mass balance in adiabatic melting of a pyroxenite-bearing peridotite. *Geochem. Geophys. Geosyst.* **16,** 267–300 (2015).
